# A Structured Cleaving Mesh for Bioheat Transfer Application

**DOI:** 10.1109/OJEMB.2020.2994557

**Published:** 2020-05-14

**Authors:** Rohan Amare, Amir A. Bahadori, Steven Eckels

**Affiliations:** ^1^ Institute for Environmental Research and Alan Levin Department of Mechanical EngineeringKansas State University 5308 Manhattan KS 66502 USA; ^2^ Radiological Engineering Analysis Laboratory; ^3^ Alan Levin Department of Mechanical and Nuclear Engineering at Kansas State University 5308 Manhattan KS 66502 USA; ^4^ Institute for Environmental Research; ^5^ Alan Levin Department of Mechanical and Nuclear Engineering at Kansas State University 5308 Manhattan KS 66502 USA

**Keywords:** Cartesian grid method, image-based modeling, finite-volume method, human thermal modeling, voxel-based mesh, volumetric mesh

## Abstract

*Goal:* The thermoregulation mechanism is a complex system that executes vital processes in the human body. Various models have been proposed to simulate the thermoregulatory response of an adult human to environmental stimuli. However, these models generally rely on stylized phantoms that lack the anatomical details of voxel phantoms used in radiation dosimetry and shielding research. The goal of this work is to introduce voxel phantoms to thermoregulation research by modeling the physical energy exchange between tissue and its surroundings, discuss a specific challenge associated with voxel phantoms, propose a method to address this challenge, and demonstrate its application. *Method:* One of the major challenges in using voxel phantoms is the stair-step effect on the surface of the voxelized domain. This effect causes over-estimation of surface area, accurate knowledge of which is critical for modeling heat exchanging systems. A methodology to generate a voxel domain from medical imaging data and reduce error in the surface area caused by the stair-step effect is presented. The methodology, based on a structured mesh and finite-volume method, is demonstrated with tumors generated from magnetic resonance imaging (MRI) scans of mice. *Results:* The methodology discussed in the paper shows a decrease in surface area over-estimation from 50% to 15% for a sphere and 47% to 17% for tumor models generated directly from MRI scans. *Conclusion:* This work provides a direct method to generate a smoother domain from medical imaging data and reducing surface area error in a voxelized domain. The technique presented is independent of domain material, including tissue type, and can be extended to any homogeneous or inhomogeneous domain. The increase in surface area accuracy obtained by smoothing the voxel domain results in more accurate temperature estimates in heat transfer simulation.

## Nomenclature

*A*
: Cross-section Area

${A_s}$

: Surface Area
***A***
: Coefficient matrix made using *UA*
***B***
: Right hand side matrix of the equation consisting of constants
*R*
: Outermost radius of the sphere
*T*
: Temperature at the point

${T_{amb}}$

: Ambient temperature

${T_b}$

: Blood temperature

${T_s}$

: Surface Temperature of the domain

${T_t}$

: Tissue temperature

${{\Delta }}{T_{er}}$

: Temperature error

$T_i^j$

: Temperature at *i*^th^ mesh element at *j*^th^ timestep
*UA*
: Overall heat transfer coefficient
*V*
: Volume of the domain
*X*
: Temperature matrix

$c_{b}$

: Specific heat of blood

$c_{t}$

: Specific heat of tissue
*e*
: Error
*h*
: Convective heat transfer coefficient
*k*
: Thermal conductivity of the domain material

$\dot q$

: Heat generation rate

${\dot q_m}$

: Metabolic heat generation rate
*r*
: Radii value in a sphere less than *R*
*s*
: Generic representation of x, y, z axes
*t*
: Time
*x*
: x-axis
*y*
: y-axis
*z*
: z-axis

*Greek alphabets*

${\nabla ^2}$

: Laplacian Operator

${\omega _b}$

: Blood perfusion rate

${\rho _t}$

: Density of tissue

## I. Introduction

The human body executes a complex control scheme in an attempt to maintain a constant core temperature. The hypothalamus acts as a thermostat that receives input signals from different parts of the body and responds with different control mechanisms to regulate heat exchange and create a homeothermic core. This entire process of receiving input signals and providing feedback response is called the human thermoregulation mechanism. Thermophysiological models such as the Fiala model [1], [2] are used to simulate this process to understand the response of the human body to its thermal surroundings. The Fiala and other models used in thermophysiological and human thermal comfort studies rely on stylized phantoms [3].

A parallel branch of research that deals with biomedical applications [4] and radiation dosimetry [5]–[7] makes use of computational human phantoms (CHPs). CHPs initially consisted of stylized phantoms, which were made using simple geometrical objects. With advancements in technology and available resources, the stylized phantoms were largely replaced by voxel phantoms and later by a family of hybrid phantoms. Voxel phantoms are generated from medical imaging data and provide a detailed and accurate representation of human anatomy. Hybrid phantoms are constructed using non-uniform rational B-spline (NURBS) surfaces and polygon mesh elements that provide a smooth surface for organs and the entire human body.

Voxel phantoms present the challenge of a stair-step effect on curved surfaces, especially for smaller organs like eye balls, due to the voxel shape [4]. Tetrahedral mesh-based (TM-based) CHPs are used as a solution to overcome the challenges of hybrid and voxel phantoms [4], [8].

The present study focuses on using a structured form of the cleaving mesh to (1) generate a simulation domain directly from medical imaging data of the patient and (2) perform heat-transfer simulations using this domain. This method makes generation of a mesh from medical imaging data straightforward, without any need for conversion to other phantom surface representations [4], [8].

The remainder of the paper is organized into sections that outline the proposed methodology and its significance. Section II provides a short review of models used in human thermophysiological research. Challenges associated with use of these models point to the need for a model that is based on accurate representation of human anatomy.

## II. Thermophysiological Models

**Fig. 1.**
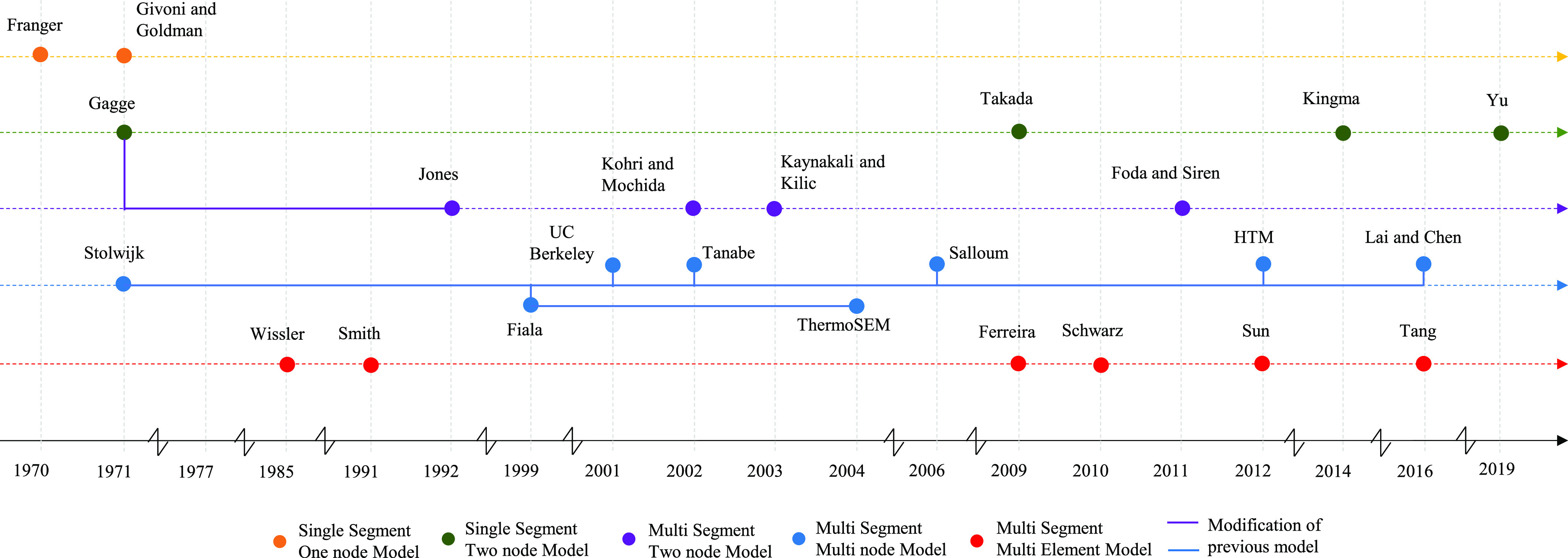
Timeline of thermophysiological models.

**Fig. 2.**
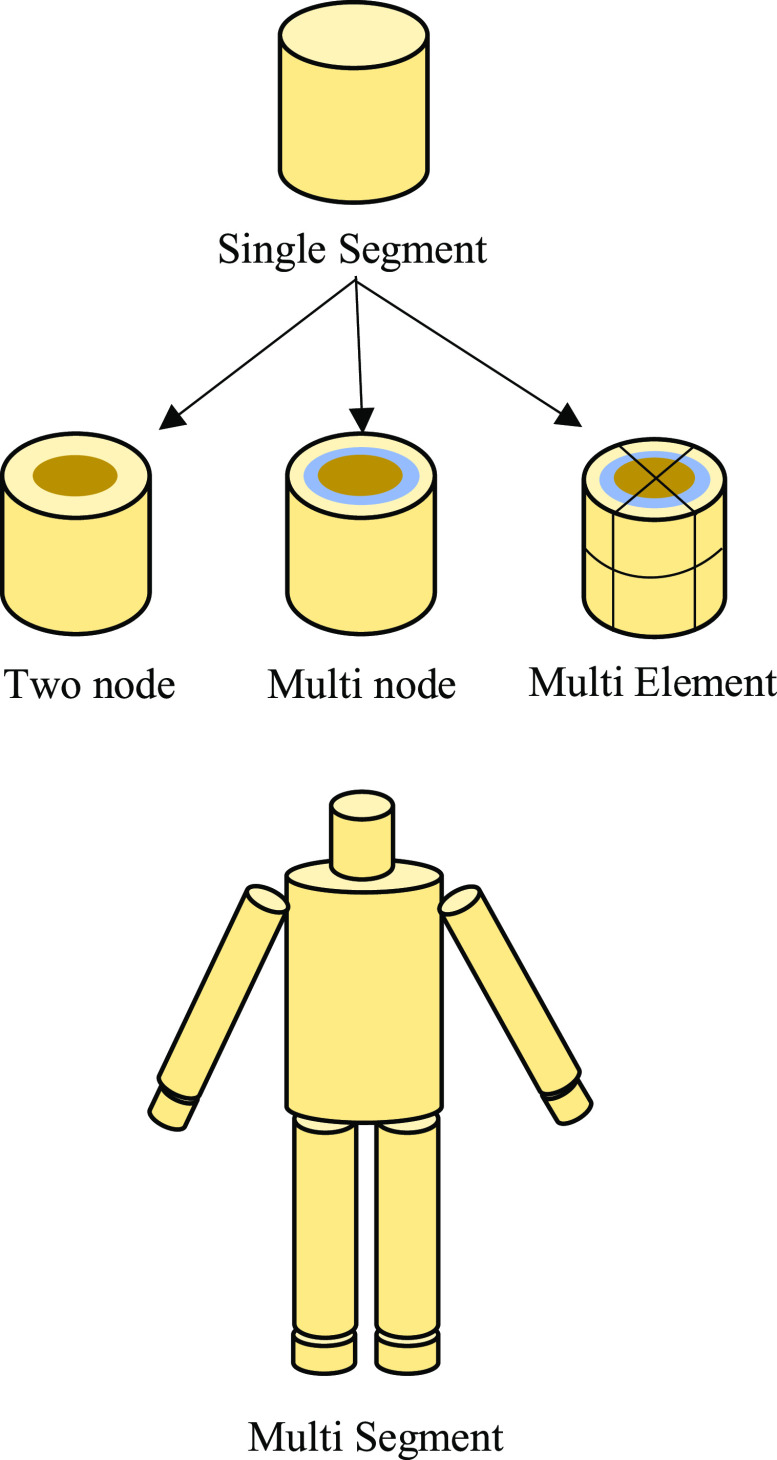
Classification of thermophysiological models.

Thermophysiological models consist of two main components: the simulation domain, which is the human body, and control equations. Control equations are used to determine sweat rate, shiver rate, changes in respiration rate, and vasomotion as a function of core temperature, skin temperature, ambient temperature, and other surrounding environmental parameters such as humidity and thermal radiation. Control equations, when coupled with the human anatomy domain for simulation, provide the complete thermophysiological model. This anatomical simulation domain representation has undergone various changes and modifications to better represent the human body. Classifications of various thermophysiological models, based on representation of the human body, are shown in a timeline in Figure 1 and classified into five different categories as shown in Figure 2. Elaborations of these classifications follow.

### A. Single Segment Models

Single-segment models consider the body as a lumped object and do not provide a detailed thermal distribution within the anatomy. They are further divided into one-node or multi-node models, based on the location and number of nodes where temperature measurement is determined.

#### 1) One-Node Model or Empirical Models

One-node models are empirical models that consider the entire human body as one lumped object exposed to the environment. Franger, and Givoni and Goldman are examples of these models [9]. These models are generated by collecting data on the human thermoregulatory reaction to environmental conditions and fitting a mathematical model. The empirical models are thus applicable only if the exact conditions of the environment and the subject are met. With variations of environmental and biological conditions of the subject, empirical models face challenges if those conditions were not considered when the model was developed.

#### 2) Two-Node Model

Empirical models provide a database of the general response of the human body to certain environmental conditions. The need for a detailed thermal audit provided more insight into thermoregulation mechanisms within the human body, resulting in single-segment, two-node models. Gagge [10] developed the first single-segment, two-node model, which has undergone many modifications. Takada [3], Kingma [3], and Yu [11] are other examples of single-segment two-node models.

These models divide the human body in a shell-and-core arrangement where the shell represents the skin layer. Such models helped to determine the distribution of blood flow between the core and skin. However, intricacies due to the shape of the entire human body and organ placement within the core are not covered in these models.

### B. Multi-Segment Models

Limitations of single-segment models were overcome by multi-segment models by providing different segments allocated to represent different parts of the human body. These models can be further classified as follows:

#### 1) Two-Node Model

One of the most basic representations of multi-segment, two-node model is the Jones model [12], which is a modification of the Gagge model [10]. The Jones model retains the shell and core structure while dividing the skin further into multiple segments to represent skin over different parts of the body such as head, torso, arms, and legs. Other models shown in Figure 1, which belong to multi-segment two-node models, have similar arrangements [3]. One of the latest models developed by Yu *et al.* [11] uses a two-node model coupled with a nonlinear heart-rate regulation model to determine human thermal behavior.

#### 2) Multi-Node Model

The multi-node model and the multi-element model are the two most developed and detailed categories of the human thermal model family. The Stolwijk model [13] provided the foundation for all other models in this family. Stolwijk divided the human body into 25 nodes and six segments to predict the thermal response of astronauts in outer space. A central blood pool was used to act as the heart from which the blood circulates to different nodes across the body. The Stolwijk model does not consider crossflow heat exchange between arteries and veins, nor does it consider variation in blood flow and other tissue properties locally. This model, however, provided a foundation for highly detailed models like Fiala [1], [2], [14], Tanabe [15], Salloum [16], Lai and Chen [17], and others. The Fiala model is one of the state-of-the-art models used to represent human thermoregulation for subjects exposed to different environments. Tanabe modified the Stolwijk model by increasing the number of nodes from 25 to 65 with 16 body segments. CFD application of the model is shown in [15]. The Lai and Chen [17] model is based on the Fiala model and modifies it to consider the non-uniform thermal environment surrounding the human subject.

#### 3) Multi-Element Model

Multi-element models are similar to multi-node models, with the major difference being that each element is constrained to have only one location where the quantity is measured. In other words, multi-node models have sub-layers or sub-sections that divide the element further, whereas in multi-element model, there are no such sub-divisions within the element. The Wissler model [18] is one of the most developed multi-element models, consisting of a detailed blood-flow network based on Pennes’ bioheat equation [19]. The Smith model [20] and Suns model [21] are based on Wissler. While Wissler and Smith used cylinders to represent the anatomy, Sun introduced curvature on the cylinders to have a more accurate representation of human limbs.

### C. Using Voxel Phantoms for Human Thermoregulation

The study of thermoregulation research brings forth the fact that all models used are stylized, incorporating geometrical objects such as cylinders and spheres to represent the human anatomy, except Tanabe [15], who uses surface points to create a manikin in the simulation domain. Stylized phantoms are readily modified to account for inter-individual in body height, weight, and organ size, as organs are represented by simple geometrical objects. However, stylized models do not provide detailed representations of human anatomy. Voxel phantoms are usually generated from medical imaging data, and thus they better represent human anatomy. The goal of the current research is to develop a human thermoregulation model that incorporates detailed representation of human anatomy and can be applied to phantoms generated from medical imaging data for specific individuals. To achieve this, voxel phantoms used in the field of computational human phantoms (CHP) for biomedical applications [4] and radiation dosimetry research [5]–[7] are used to represent the human body.

**Fig. 3.**
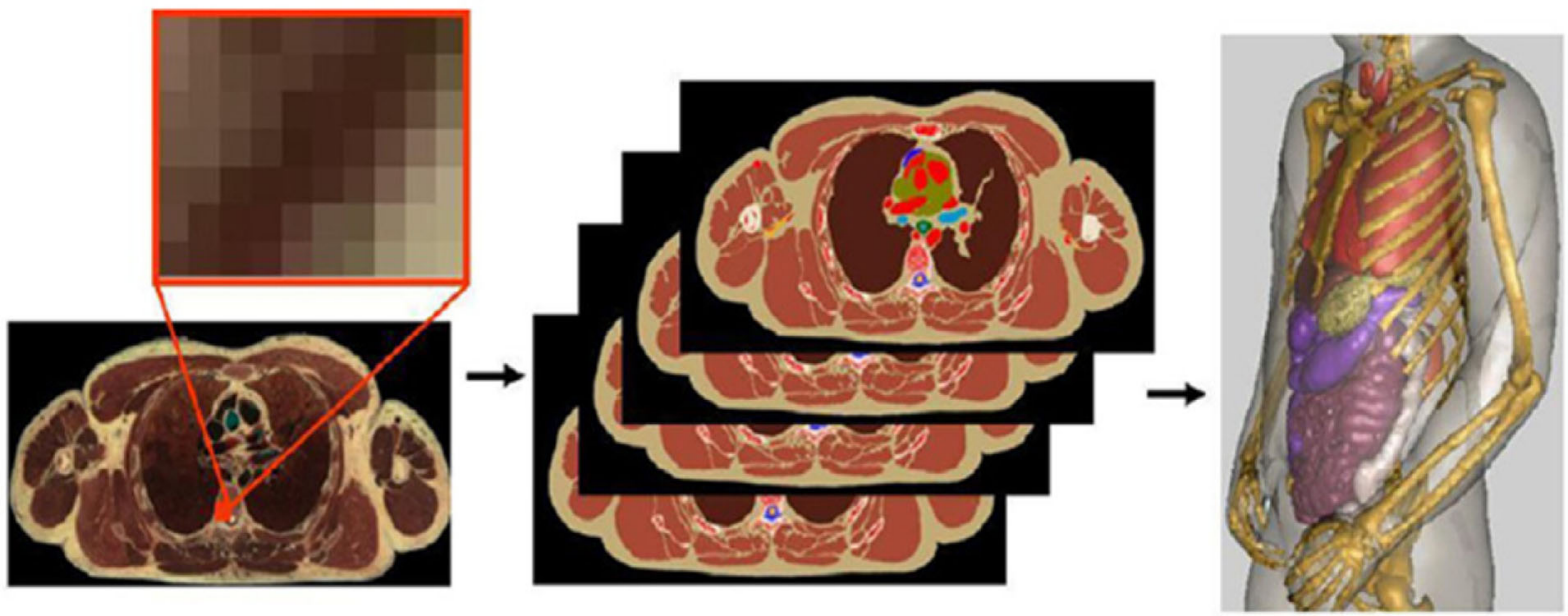
Computational human phantoms — step-by-step procedure to create a voxel phantom (used with permission from [19]).

Voxel phantoms are generated from medical imaging such as computed tomography (CT) or MRI scans. Axially continuous medical imaging data are collected, and pixels are extruded to the thickness of the slice. Figure 3 shows the step-by-step development of voxel phantoms from medical imaging data.

### D. Challenges of Using Voxel Phantoms

Voxel phantoms rely on segmentation of medical imaging data to sort organs and tissue from each other. This process still relies on manual segmentation for accuracy in representing organs and tissues. However, automatic segmentation algorithms to convert CT values to tissues are being developed [22]. Voxel phantoms can be generated using detailed high-definition color photographs that permit more accurate segmentation compared to CT scans. One such example is the high-definition reference Korean-man (HDRK-Man) [23].

The next challenge with the voxel phantom is the cuboidal nature of the voxel [24], [25]. The cuboidal structure results in a stair-step effect on curved surfaces, producing organs with rough surfaces [26]–[28]. The stair-step effect causes overestimation of surface area [29], [30], which is significant when considering surface area-dependent heat transfer processes such as convection and radiation.

To address the challenge posed by the cuboidal shape, various methods have been proposed. Converting voxel phantoms to polygon mesh phantoms [4], [31] provides better accuracy in surface area, but at the cost of increased computational resources. Tetrahedral mesh-based phantoms, developed by Yeom *et al.* [8], are also used as they provide a better computational time than polygon mesh. Polygon mesh elements can have more than six facets. Thus, more computational resources are required to store information and perform calculations on every face of the element. Though polygon meshes provide exceptional representations of complex surfaces, the computational requirements pose a challenge for simulation. Thus, polygon meshes can be converted to tetrahedral meshes as the mesh element is reduced to have only four faces, reducing the computational requirements significantly.

Samaras *et al.* [30] proposed correcting the heat-transfer coefficient value by using the ratio of surface-area overestimation to actual area to compensate for the stair-step error. However, this method works only when the actual area of the organ or the individual subject involved in the simulation is known. The value of the heat-transfer coefficient would thus be different for each individual after using the correction factor. The uncertainties and challenges one could encounter in measuring the actual surface area of each individual subject need to be investigated further. Dillard *et al.* [32] developed a framework to generate a smooth 3D domain from a Cartesian grid. The framework faces challenges when modeling multiple organs using medical imaging data.

A similar algorithm based on the marching cube method [27] is used to model two adult and two children phantoms [28]. The marching cube method is by far one of the most-used algorithms to smooth a surface represented by cuboidal structures [33]. This method converts the outer layer to a tetrahedral mesh while the internal elements are still hexahedral. In the marching cube method, a scalar value is stored at the lattice point. This scalar value is the pixel information in case of an image. Each lattice point is a corner vertex of a cube and eight such lattice points defines one cubic voxel. This arrangement of information on the vertices of the cube helps to generate the intersection topologies which results in smooth surfaces.

Pixels obtained from imaging data can be easily extended to a voxel in 3D with the information stored in the center of the voxel. A voxel domain generated this way can be easily used for finite-volume analysis. Since the finite volume method (FVM) uses flux balance across the faces, the equations are more straightforward and easier to handle for simulation compared to those of the finite element method (FEM). However, surface-smoothing algorithms used for an FEM mesh cannot be used for an FVM mesh and thus methods such as a marching cube face a challenge. Compared to simulations and models that use FEM, very few models can be found that use FVM for medical applications. Crockett *et al.* [34] is an example that uses FVM. They use the volume of fluid (VOF) method to smooth the surface under consideration for simulation.

Lattice cleaving, developed by Bronson *et al.* [35], is another method to smooth an interface of a voxel domain. The method generates a tetrahedral mesh to approximately conform to the surface area interface between materials. The method cuts a body-centered cubic lattice to match the surface, similar to the marching cube method. The generated mesh is tetrahedral, unstructured, and allows mesh element sizes to vary, thus having fewer elements in the region where an interface is not present. The method uses 24 stencil tetrahedrons that vary based on the cuts on the lattice.

The present paper aims to generate a structured tetrahedral mesh using a method similar to lattice cleaving that can smooth a material interface in a voxel domain. The proposed method divides the lattice into 24 tetrahedrons. By generating mirror images along different axes, the 24 tetrahedrons are produced from a single reference tetrahedron. The methodology to smooth the interface, and its effect on reference surface area and volume, are described. The method is used on a sphere as a benchmark heat-transfer simulation. The methodology is applied to four tumors obtained from MRI scans of mice and heat transfer simulations are performed.

## III. Methodology

Previous work [29] introduced the technique of using the FVM for a 2D domain generated from medical imaging data. It introduced a technique to decrease surface area overestimation from 27% to 5% for a circle in 2D. The present work is an extension of [29] from a 2D pixel domain to a 3D voxel domain. The methodology is divided into three major steps, which consist of pre-processing, converting voxels to tetrahedrons, and smoothing.

### A. Step 1: Pre-processing: Determining Surface Voxels

Voxels in the voxel phantom carry different tags. Depending upon the type of tag, they can represent the voxel material or different properties of the voxel. Along with these existing voxel tags, a new tag is created that will differentiate a voxel on the surface of the domain from a voxel on the interior. This tag will be termed the “side-exposed tag” for the rest of this paper. The side-exposed tag stores information about which sides of the voxel are exposed to different materials. This information will be used to smooth the surface in the following steps. Once all of the voxels are given a tag to represent the sides exposed, they will be converted to tetrahedrons.

### B. Step 2: Converting Voxels to Tetrahedrons

Previous work [29] divides a 2D domain of square pixels to triangles. The approach is extended by dividing each cuboidal voxel into 24 tetrahedrons.

**Fig. 4.**
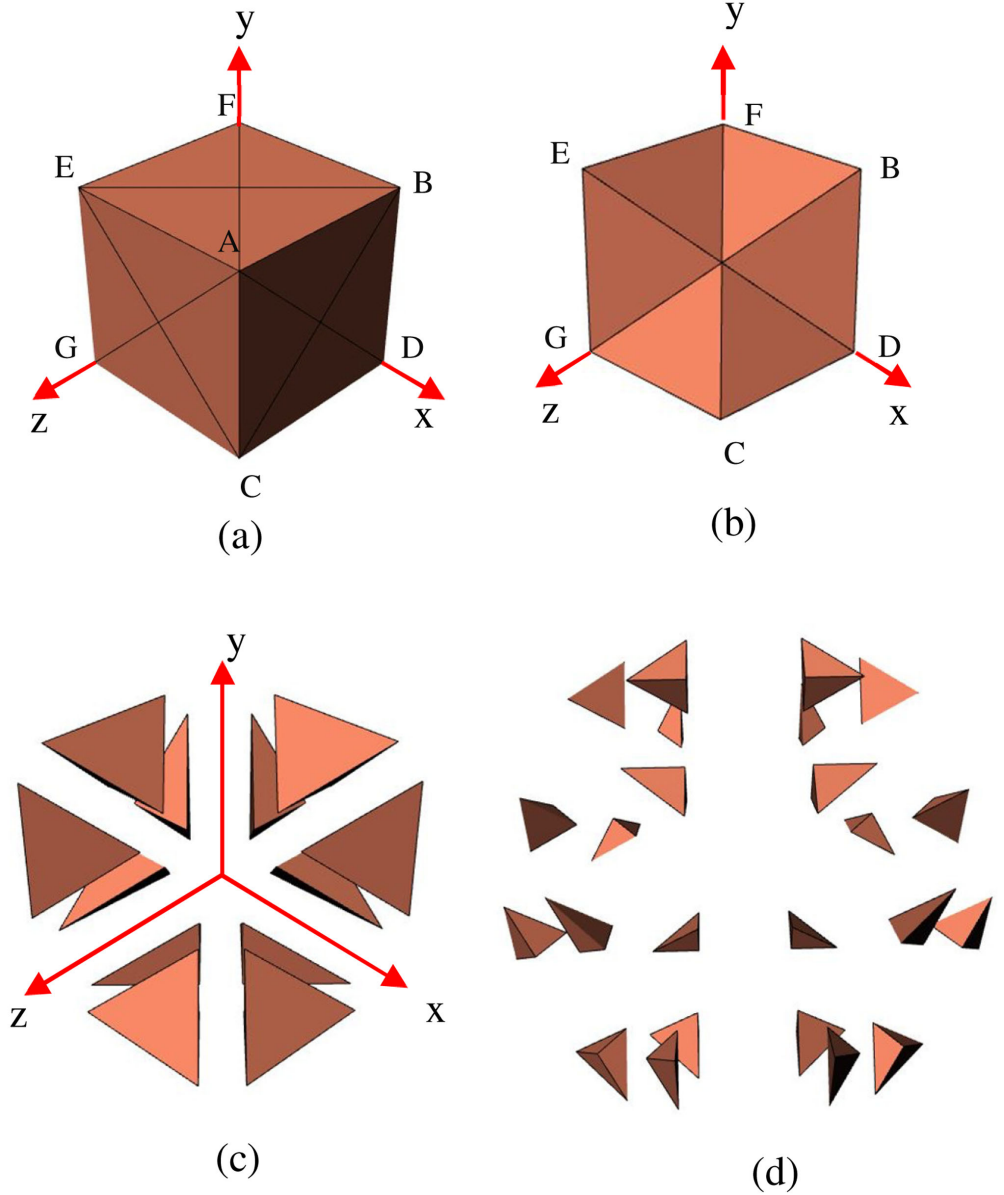
Voxel to tetrahedron (a) voxel with the diagonals to cut along, (b) a section of the voxel generated using tetrahedrons, (c) exploded view of tetrahedrons, and (d) exploded view of all 24 tetrahedrons.

Consider a voxel shown in Figure 4(a), which is cut along all the diagonals shown by AF, BE, EC, AG, BC, and AD on all sides. The cut along the diagonals results in 24 tetrahedrons such that they share a common vertex with the centroid of the voxel. Each side is now represented with four voxels. Figure 4(b) shows the four tetrahedrons for a section of the voxel. The exploded view of Figure 4(b), showing the tetrahedrons separated, is in Figure 4(c), and all 24 tetrahedrons are shown in Figure 4(d) as an exploded view. Each of these tetrahedrons has the same material as that of the parent voxel.

### C. Step 3: Smoothing the Surface

Converting the voxels to tetrahedrons alone does not solve the problem of the stair-step effect on the surface. Thus, a smoothing algorithm is needed to smooth the surface of the object after converting voxels to tetrahedrons.

**Fig. 5.**
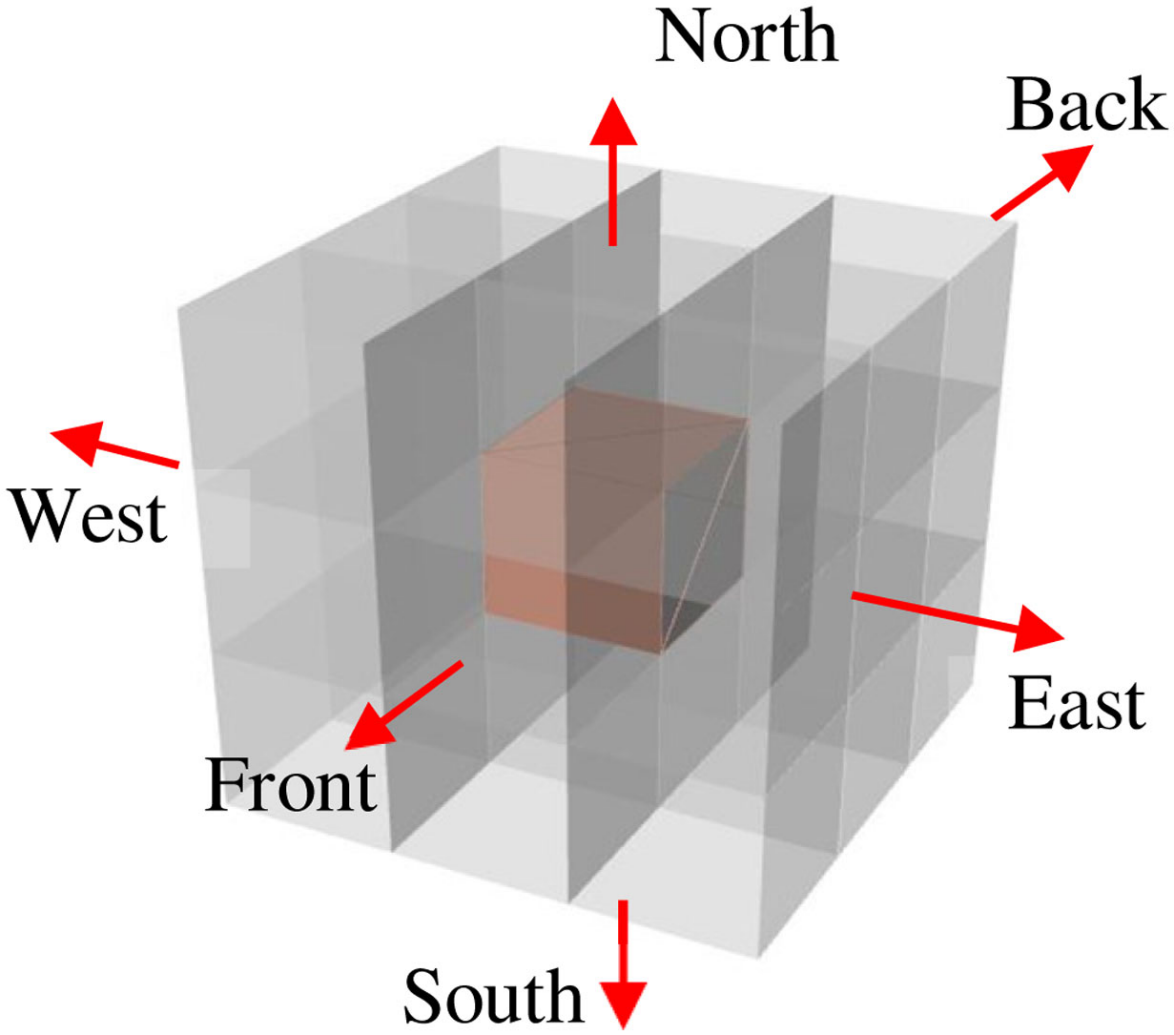
Voxel array. The salmon color voxel represents the voxel under consideration. The grey voxels represent the surrounding voxels.

**Fig. 6.**
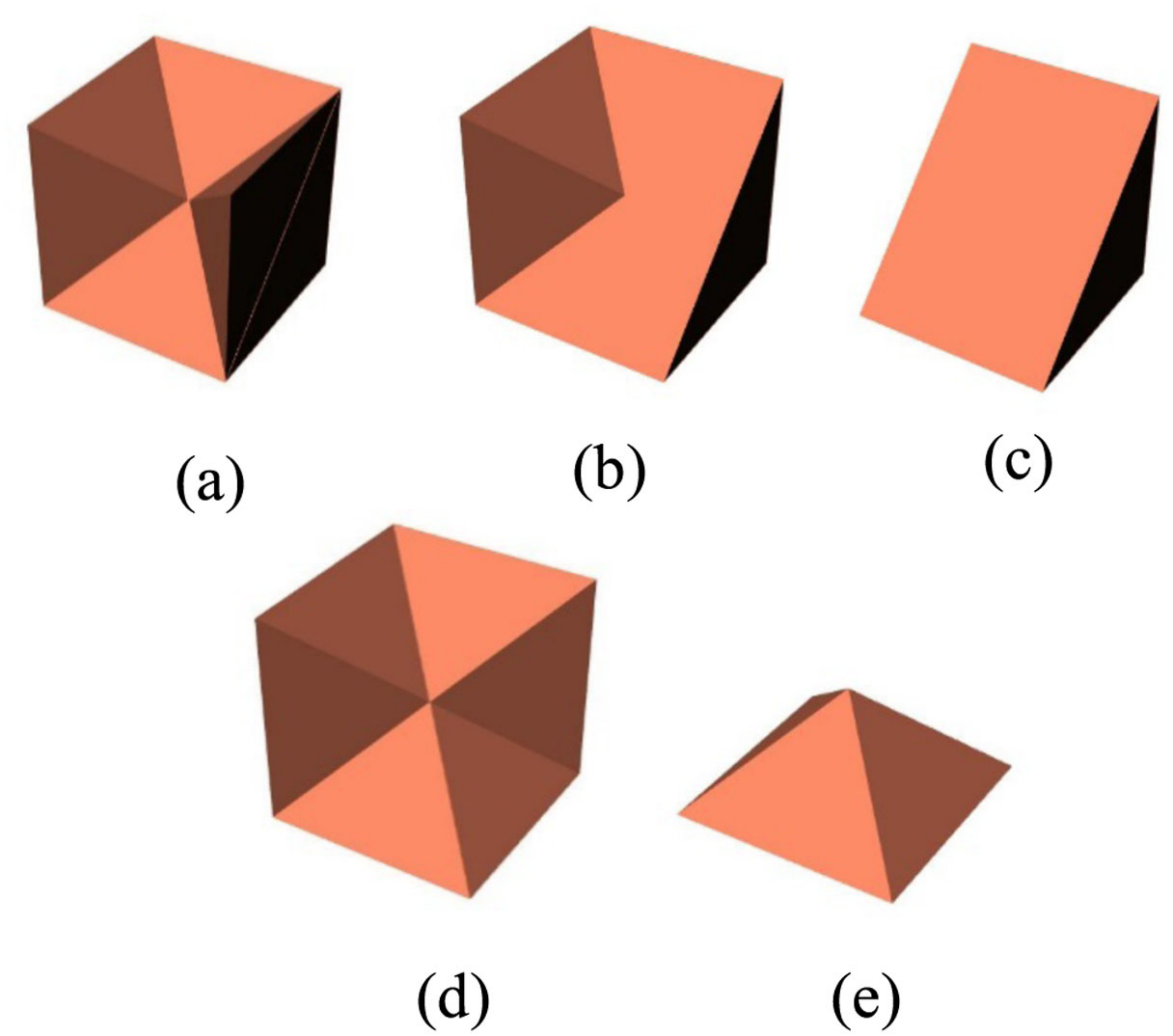
Modification of parent voxel.

**Fig. 7.**
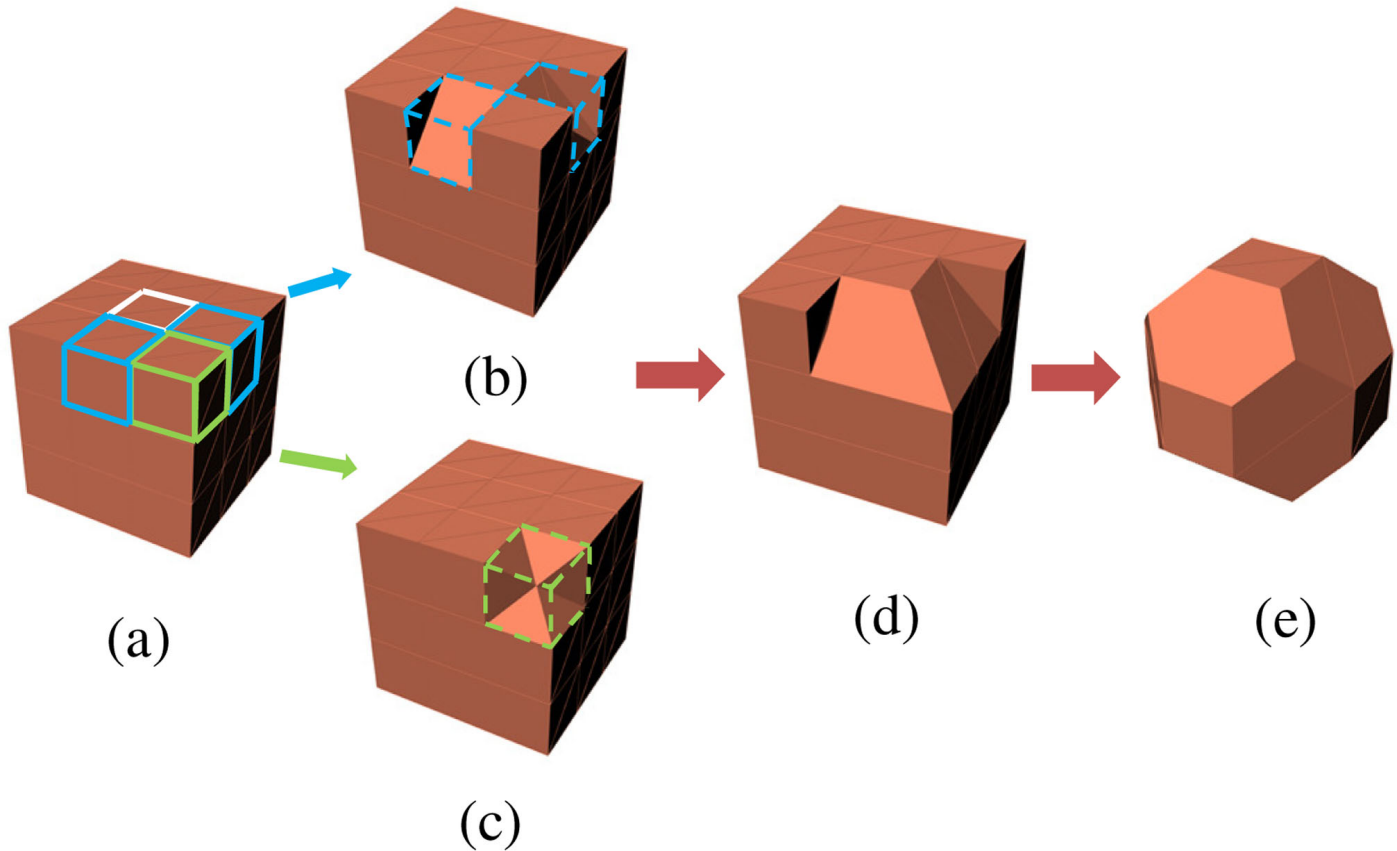
Methodology to smooth the domain. (a) unsmoothed 3 × 3 × 3 grid of voxels. White outline shows one side exposed voxel, blue outline shows two side exposed voxel and green outline shows a three-side exposed voxel. (b) Smoothing of two side exposed voxels. (c) Smoothing of three side exposed voxel. (d) Two-side and three-side exposed voxels smoothed on one vertex. (e) End result of smoothing.

Consider the parent voxel shown in Figure 5*,* surrounded with neighboring parent voxels. The red arrows show the six directions with reference to the voxel under consideration. Figure 6 shows the various modification a voxel undergoes during the smoothing process. Figure 7 represents a matrix 3 × 3 × 3 of voxels where (a) represents an unsmoothed domain with one-side, two-sides and three-sides exposed voxels marked in white, blue and green outlines for reference respectively. The entire domain is made up of same material and is exposed to surrounding material, which is not shown. The smoothing procedure is demonstrated in Figure 5, Figure 6 and Figure 7, and is described in the following steps.

1. If the parent voxel has no side or only one side exposed to different material, then the voxel does not undergo modification. In Figure 7(a), the voxel marked with a white outline is shown as an example of such a voxel with only one side exposed to different material. This voxel will not be changed.
2. If parent voxel has more than one neighboring voxel of different material, for example, north and front voxels, the material tags of the tetrahedrons, which are part of the side exposed to these voxels, will be changed from parent material to neighboring voxel material. The parent voxel after modification is shown in Figure 6(a).
3. The side-exposed tag of neighboring voxels will now be checked. If the east neighboring voxel has more than one side exposed to a different material, even the neighboring voxel will be modified. Thus, the material tags of tetrahedrons that share the edge with east-north and east-front sides, respectively, are also changed. The result is shown in Figure 6(b).
4. Similarly, if the side-exposed tag of west-neighboring voxels shows more than one neighbor of different material, the material tags of tetrahedrons that share the edge with the west-north and west-front sides, respectively, are changed. This modification result is shown in Figure 6(c). A smooth diagonal cut of the parent voxel under consideration is produced. The result of steps 2, 3 and 4 is shown in Figure 7 as a transformation from Figure 7(a) to Figure 7(b). The two-side exposed voxels marked in blue in Figure 7(a) result in the diagonal cut voxels as shown in Figure 7(b)
5. Consider a voxel with north, front, and east sides exposed to neighbors, i.e. three sides exposed. Similar to the two-sides-exposed condition, material tags of the tetrahedrons that are part of the voxel's three sides will be changed to material of the neighboring voxels in the respective direction. In other words, the material of these tetrahedrons will be changed to that of the surrounding material to the domain. The resultant parent voxel structure is shown in Figure 6(c). In Figure 7(a), the three sides exposed voxel is shown on a vertex marked by a green outline. This voxel after undergoing the modification is transformed in a shape as shown in Figure 7(c). When all the voxels closer to each other are modified, a partially smoothed domain is similar to one shown in Figure 7(d). Figure 7(d) shows the result of two-sides exposed voxel modification and three-sides exposed modification on one corner of the grid.
6. For a case where five sides are exposed to different materials, the resultant parent voxel is a pyramid with one side that is not exposed to a different material. This is shown in Figure 6(e). A five-sides-exposed voxel will be completely converted to the neighboring material, as the surface area of a pyramid is greater than one side of a cube.
7. The end result of applying the smoothing algorithm on the grid shown in Figure 7(a). results in a domain shown in Figure 7(e).

### D. Smoothing Demonstration With Medical Imaging Data

**Fig. 8.**
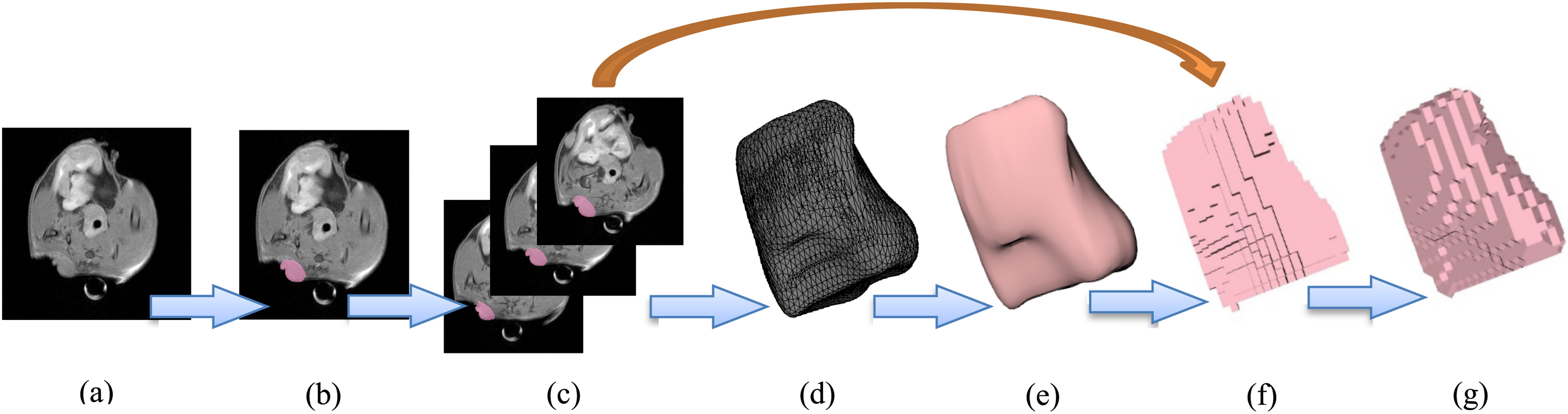
The process to generate a tumor model from an MRI scan and smooth it is shown by the blue arrows. The golden arrow shows that mesh and NURBS objects can be avoided to use the proposed methodology.

To demonstrate the described methodology, MRI scans of four lab mice with tumors were obtained from the Biomedical Computing and Devices Laboratory at Kansas State University. The tumors in these scans were manually segmented using 3DSlicer (version 4.8.1) [36]. The voxel size obtained from these scans was 0.117 × 0.117 × 1.5 mm. A 3D unstructured mesh of each tumor was generated using the 3DSlicer meshing feature. This mesh was converted to a NURBS surface using Rhinoceros (version 5) [37] to obtain a reference surface area and volume for determining the accuracy in these quantities for this methodology. The domain was re-voxelized with cuboidal voxels with the same dimensions in all three axes. This process is described in Figure 8, which shows, using blue arrows, development from an MRI scan to the final, tetrahedral smoothed tumor undertaken for this paper. The golden arrow from Figure 8(c) to Figure 8(f) indicates that though a NURBS surface was generated in this paper to use as a reference, there is no need to generate a NURBS surface to apply the proposed method to a medical imaging dataset. To show the effect of this method on simple geometric objects, a sphere and cylinder were also considered. The volume and surface area convergence for a sphere with a radius of 1 cm, and a cylinder with a radius of 1 cm and height of 2 cm are demonstrated along with the tumors.

### E. Heat Transfer Simulation

The Pennes Bioheat Equation [19] shown in Equation (1) is used to model heat transfer in many biological simulation domains and thermophysiological models.

\begin{equation*}
{\rho _T}{c_{T\;}}\frac{{\partial T}}{{\partial t}}\; = {\nabla ^2}\left({kT} \right) + {\omega _b}{c_b}\left({{T_b} - T} \right) + \dot q\tag{1}
\end{equation*}

In Equation (1), the left-hand side represents the rate of energy storage per unit volume and the terms on the right-hand side represent heat transfer due to conduction, heat dissipation due to blood flow, and heat generation rate, in the order shown. The blood perfusion term depends on the blood perfusion rate ${\omega _b}\;$ for the tissue under given conditions and the temperature difference between the arterial blood and the tissue temperature. Many criticisms have been raised for the Pennes perfusion term and its application [38] mainly due to the difficulties of measuring the blood perfusion rate in-vivo for different tissues. The heat generation rate can consist of metabolic heat rate and exogenous energy deposition rate.

In the present study, the following assumptions are made for simplicity. The blood perfusion rate is assumed to be zero. The system is solved for a steady state condition and there is no heat generation source except metabolic heat generation within the tissue. With these assumptions, the Pennes equation is reduced to the generic steady state conduction equation with heat generation as shown in Equation (2).

\begin{equation*}
{\nabla ^2}\left({kT} \right) + \dot q = 0\tag{2}
\end{equation*}

The discretization equation presented in previous work [29] for a 2D domain is expanded for a 3D domain as shown in Equation (3)

\begin{equation*}
\mathop \sum \limits_{i = 1}^n {\left({UA} \right)_i}\left({{T_i} - {T_0}} \right) + \dot q{\rm{d}}V = 0\tag{3}
\end{equation*}

where

\begin{align*}
\frac{1}{{{{\left({UA} \right)}_i}}} &= \frac{1}{{{A_i}}}\left({\frac{{{\rm{d}}s}}{{2{k_i}}} + \frac{{{\rm{d}}s}}{{2{k_0}}}} \right)\tag{3.a}\\
\frac{1}{{{{\left({UA} \right)}_i}}} &= \frac{1}{{{A_i}}}\left({\frac{1}{h} + \frac{{{\rm{d}}s}}{{2{k_0}}}} \right)\tag{3.b}
\end{align*}

The variable *n* represents the total number of sides of the mesh element. This value of *n* is 6 for a voxel (hexahedral mesh element) and 4 for a tetrahedron. The values taken by *i* from 1 to *n* represent different directions to the mesh element and 0 is reserved for the mesh element under consideration. Thus, for a voxel mesh, 1 to 6 represent north, south, east, west, front, and back, while tetrahedron 1 to 4 represent the neighboring tetrahedrons to its four sides. The term d*s* is a generic representation of voxel dimensions, d*x*, d*y*, and d*z* in *x*, *y*, and *z* directions respectively. *A* represents the cross-sectional surface area, i.e. the area of the side under consideration. If the neighboring voxel is a fluid, and convection takes place for heat transfer, the term *UA* is calculated using Equation (3.b). The convective heat transfer coefficient is denoted by *h*.

The matrix form of the system of equations is shown in Equation (4).

\begin{equation*}
\boldsymbol{A}X = \boldsymbol{B}\tag{4}
\end{equation*}

Here, ***A*** is a sparse matrix consisting of the coefficients calculated using the *UA* values, matrix *X* represents the temperature values at mesh element, and matrix ***B*** consists of the net heat generated for each cell or boundary condition based on location of the mesh element. This makes the system of equations implicit, thus increasing the speed of computation.

The Least Square Iteration method for a sparse matrix as presented in [39] was used to solve the system of equations. The method proved most useful with respect to memory requirements and speed of computation for the simulation domains considered in this work.

### F. Benchmarking With Sphere

Benchmarking the heat transfer simulation is first completed by considering a spherical tissue domain since the analytical solution is readily available. To study the effect of voxel representation and tetrahedral smoothing, the steady-state surface temperature is calculated and compared with the exact analytical solution.

When using the removal technique for smoothing described previously, material is “lost”. Thus, it is important to find a balance between the loss of material and accurate surface representation. The difference in the volume resulting from material loss will influence total heat generated, which will affect surface temperature. To handle this effect of volume variation on surface temperature, an equation to determine the allowable tolerance in volume is derived from Newton's law of convection heat transfer. The convection heat-transfer in a domain with heat generation is shown in Equation (5)

\begin{equation*}
\dot qV = h{A_S}\left({{T_s} - {T_{amb}}} \right)\tag{5}
\end{equation*}

In Equation (5) *V* represents the total volume of the domain, $T_{s}$ and $T_{amb}$ represent the surface temperature and ambient temperature, respectively. $A_{s}$ is the surface area of the NURBS domain used as a reference area. The heat generation rate $\dot q\;$ when multiplied with the total volume of the domain *V* provides the net energy generation rate.

Rearranging Equation (5) and dividing by the reference volume *V* results in an equation that can be used to determine the volume tolerance, which is given in Equation (6):

\begin{equation*}
\frac{{{{\Delta }}V}}{V} = \left({{{\Delta }}{T_{er}}\frac{h}{{\dot q}}\frac{{{A_s}}}{V}} \right)\tag{6}
\end{equation*}

**Fig. 9.**
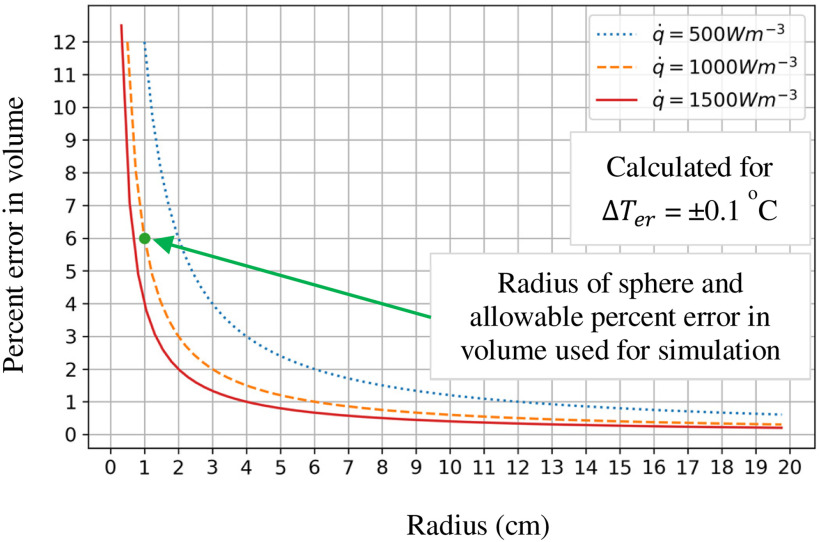
Allowable percentage volume error for temperature error $\Delta {T_{er}} = \pm {0.1^o}C$. The green dot and arrow at 1 cm radius and 6% volume error represents the value at which the simulation for sphere was conducted.

In Equation (6), ${{\Delta }}{T_{er}}$ is the allowable temperature variation due to error in the volume ${{\Delta }}V$ for the same surface area. In the present paper, this temperature variation is limited to ±0.1 ^o^C. Using this value, the allowable error in the volume ${{\Delta }}V$ is calculated. In other words, for the same surface area, the difference in volume should be within ${{\Delta }}V$ so that the variation of surface temperature due to volume difference would not be more than ±0.1 ^o^C. This allowable percent error in volume against the radius of sphere for different heat generation rates is shown in Figure 9. A convective heat transfer coefficient of 2 W/m^2^-K is used as a constant to plot Figure 9. The plot shows for a given heat transfer rate, the allowable error in volume decreases as the size of the domain under consideration increases. In this case, as the radius of the sphere increases, the allowable tolerance in volume error decreases.

Using Equation (6), the acceptable percent error for a sphere with a radius of 1 cm and a heat-generation rate of 1000 W/m^3^ is within 6%. Given that volume tolerance is inversely proportional to the heat generation rate and size of the domain, as radius increases the volume tolerance decreases for a given heat-generation rate. Thus, for a 1000 W/m^3^ and 10 cm sphere, which is an average size of the human head when approximated as a sphere, the allowable percent error on volume is 0.6%. For this paper, a sphere of 1 cm with thermal conductivity of 0.3 W/m-K was considered with 1000 W/m^3^ of heat generation rate exposed to air at 20 °C and assuming a convective heat transfer coefficient 2 W/m^2^K. This is shown in Figure 9 as a green dot at radius 1 cm and 6% volume error. The voxel size for the simulation used was 0.8 mm.

### G. Heat Transfer Application With Medical Imaging Data

Once the code was benchmarked, the heat transfer simulations for four different tumors generated from MRI scans were conducted. The MRI scans showed that these tumors were anatomically present at a subcutaneous level. A medical probe was seen in the MRI scans closer to the tumor with a small air gap between the mouse and the probe. For the simulation, the tumors are considered to be deep, meaning that they are surrounded on all sides with healthy tissue for simplicity. The resolution of the MRI scans resulted in an elongated, pin-like voxel with its length in the *z* axis that represents slice thickness. To smooth the tumor, the geometry was re-voxelized to have a cuboidal voxel with dimensions of voxel the same in all three axes. For this paper, the simulation on the tumors is performed for a voxel size of 0.12 mm × 0.12 mm × 0.12 mm. This was done to maintain the dimension of the voxel similar to the *x* axis and *y* axis resolutions of the MRI scans.

For the tumor, the metabolic heat-rate generation was considered to be 1.2 mW/mm^3^ based on the metabolic heat-production rate proposed by F. J. Gonzalez [40] for breast tumors using a non-invasive method and numerical simulation for humans. For the surrounding tissue, the metabolic heat-rate generation was considered as 0.001 mW/mm^3^ based on values obtained from the IT'IS Foundation database found online for muscle tissues [41]. The thermal conductivity for the tumor and surrounding tissue was considered to be 0.5 mW/mm-K referring to average value of tissue from IT'IS Foundation database [41]. A Dirichlet boundary condition of 30 °C was imposed on the edge of the simulation domain in all directions.

## IV. Results

**Fig. 10.**
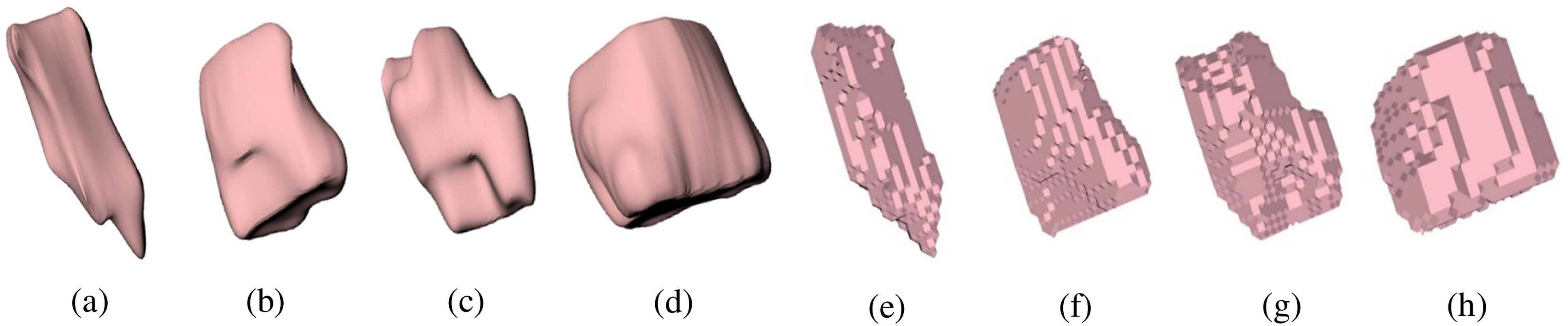
a, b, c, d — NURBS tumor models. e, f, g, h — Tetrahedral smoothed tumor.

**Fig. 11.**
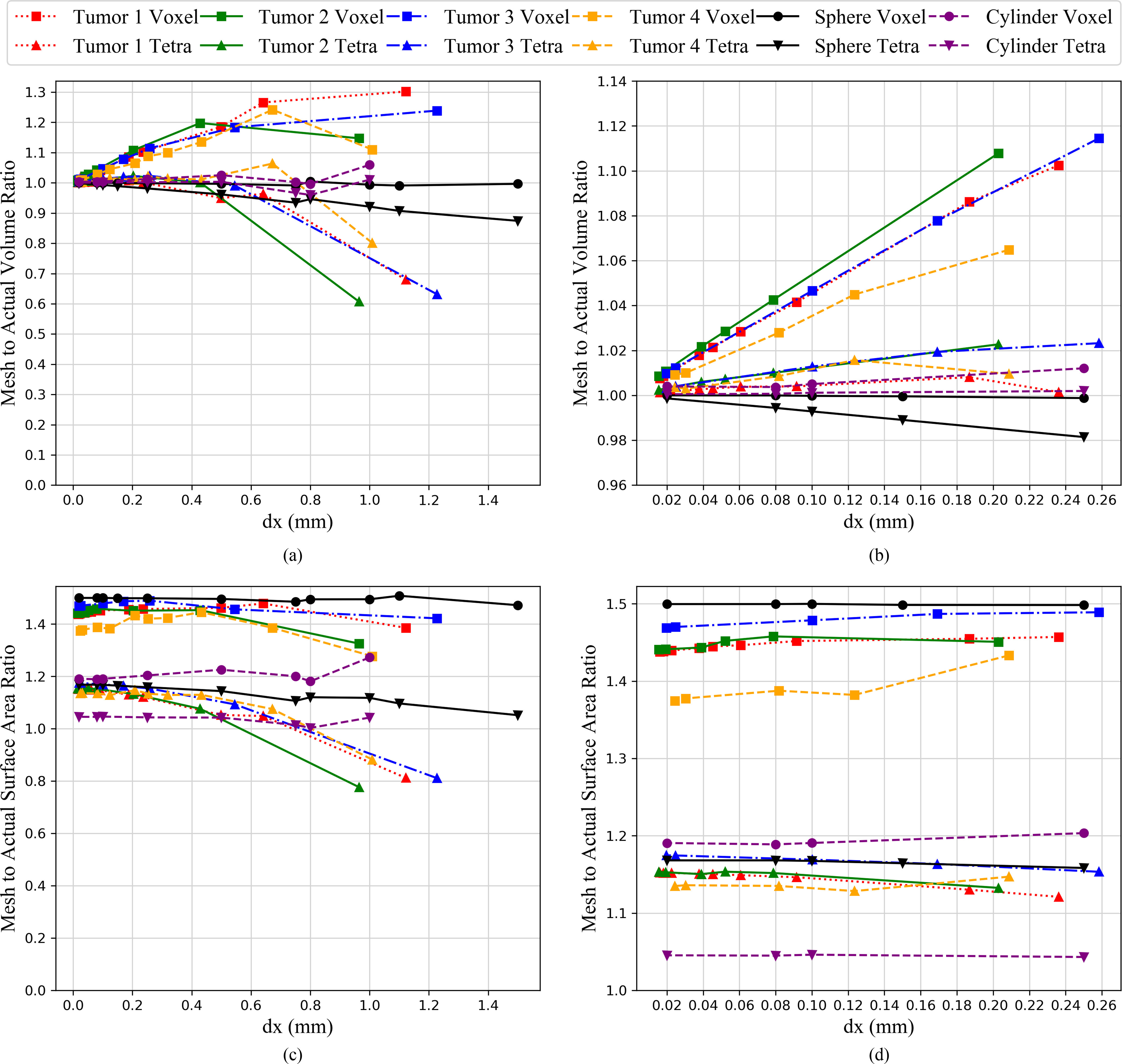
Convergence of volume and surface area.

Figure 10 shows the four tumors as NURBS and tetrahedral smoothed surfaces. The volume convergence of the four tumors, the sphere, and a cylinder are shown in Figure 11(a). The volume convergence plot shows the ratio of volume obtained from the voxel mesh or tetrahedral mesh to the NURBS volume of the object. “Perfect” volume convergence is achieved when the volume ratio is equal to one. This volume ratio is plotted against voxel size in one dimension. As the voxel size decreases, the volume rapidly converges to unity for all geometries. Figure 11(b), which is a zoomed-in representation of Figure 11(a), shows the voxel and tetrahedral mesh volume of four tumors to be within ±2% of the NURBS volume for a voxel size less than 0.05 mm; for the sphere and cylinder, this convergence is achieved for a voxel size less than 0.25 mm.

The surface-area ratio is calculated as the ratio of surface area obtained using the mesh to the surface area of the NURBS surface. Figure 11(c) shows the surface-area convergence for four tumors, a sphere, and a cylinder against the voxel sizes. Unlike the volume ratio plots where the volume-ratio for all the geometries rapidly converges to unity, the surface-area ratio plots do not converge to unity for any of the geometries. Figure 11(d), which is a zoomed-in image of Figure 11(c), shows this behavior of the surface area.

**Fig. 12.**
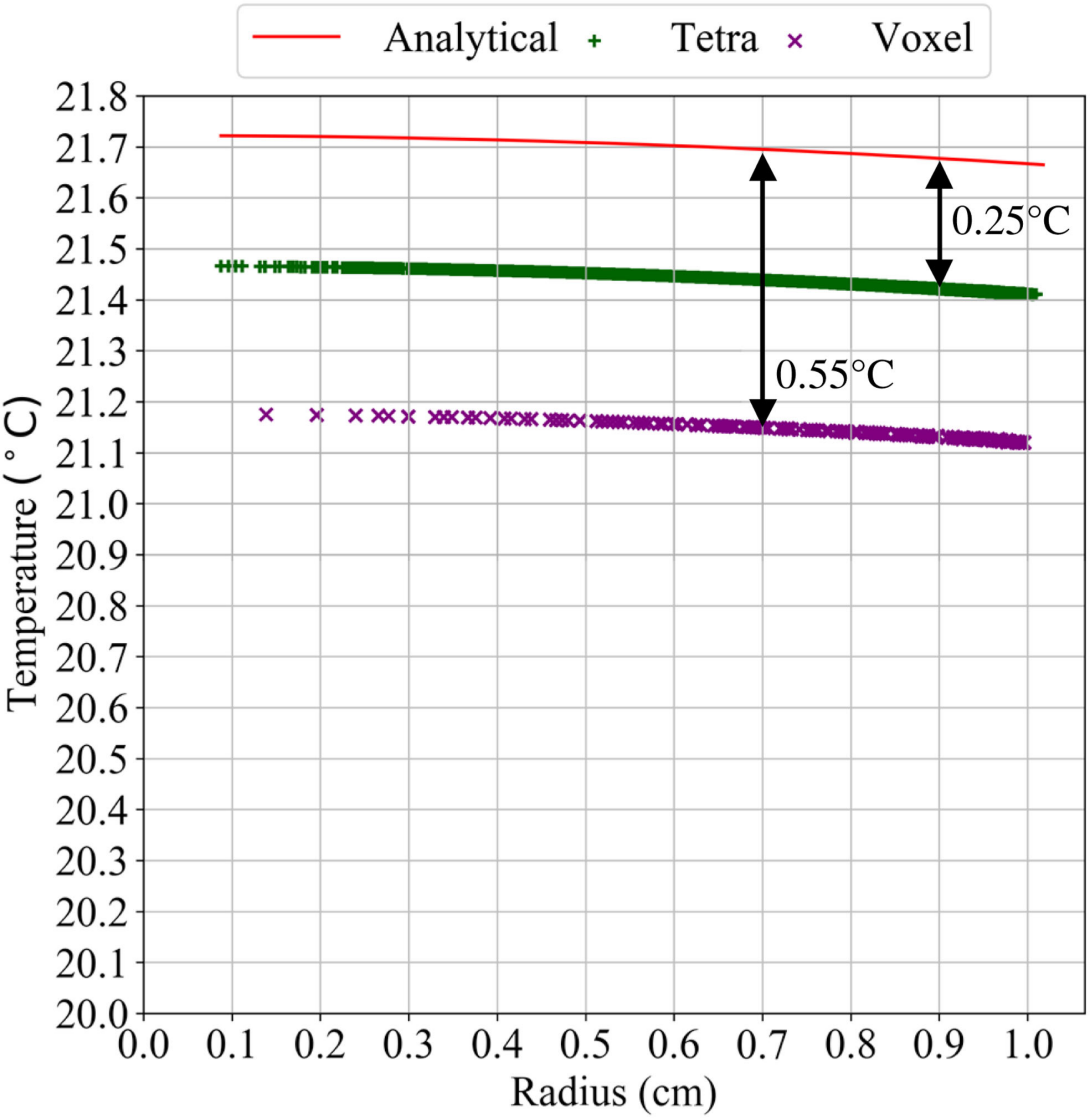
Radial temperature comparison for a sphere.

The voxel mesh sphere has a higher error than the tetrahedral smoothed mesh sphere due to greater surface area, which results in more convective heat transfer than the actual sphere. Figure 12 shows the radial temperature variation within the sphere. The temperature of each mesh element within the voxel and tetrahedral mesh for the sphere is plotted against the radial distance of the mesh from the center of the sphere. For simplicity, the radial temperature within the sphere is plotted for only an eighth section. Since the sphere is symmetric, the same temperature distribution is observed in all other sections.

The analytical solution for a sphere generating heat and exposed to air is given in Equation (7).

\begin{equation*}
T\left(r \right) - {T_{amb}} = \frac{{V\dot q}}{{{A_s}h}} + \frac{{\dot q}}{{6k}}\left({{R^2} - {r^2}} \right)\tag{7}
\end{equation*}

*V* and $A_{s}$ represent the actual volume and surface area of the sphere, and *R* represents the outer radius. This analytical solution is shown in red in Figure 12 . The error in the radial temperature shown in Figure 12 can be expressed mathematically as

\begin{equation*}
{{\Delta }}T\left(r \right) = T^{\prime}\left(r \right) - T\left(r \right) = \frac{{V\dot q}}{{h{A_s}}}\left({\frac{{1 - a}}{a}} \right)\tag{8}
\end{equation*}

In Equation (8), $a$ represents the fractional difference from the actual area of the sphere. $T^{\prime}(r)\;$ represents the radial temperature for a surface area $a$ times the actual surface area ${A_s}$. It can be seen from the Equation (8) there is an equal shift in the radial temperature which is also seen graphically in Figure 12. Thus, the temperature difference calculated at any radius is the overall temperature error at all radii.

**Fig. 13.**
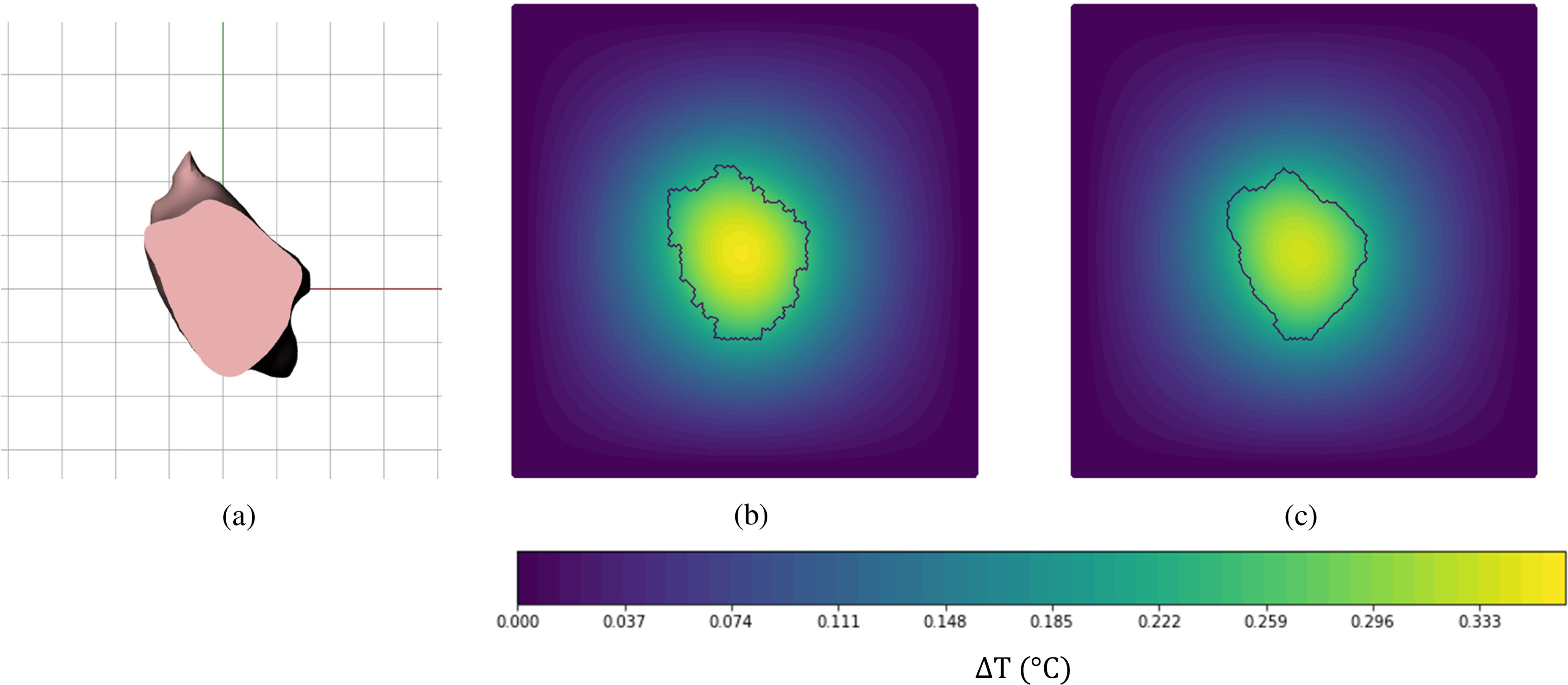
Cross-section of tumor 1 at x-y plane at midpoint of z-axial length — (a) NURBS Rhinoceros model, (b) voxel unsmoothed (c), and tetrahedral smoothed.

Figure 13 shows the cross section and thermal map for one tumor shown in Figure 10(a) as NURBS and in Figure 10(e) as tetrahedral smoothed. This tumor will be referred as tumor 1 in the following discussion. The cross section and thermal map for the other three tumors are given in the supplementary section. Figure 13(a) shows a cross section of a NURBS tumor generated using Rhinoceros software. Figure 13(b). is the temperature contour of the voxel unsmoothed tumor and Figure 13(c) is tetrahedral smoothed tumor at the same cross-section. The temperature contours plot the temperature difference $({{\rm{\Delta T}}})$ which is calculated as the difference between temperature at the given point and boundary temperature.

## V. Discussion

A sphere represents the worst possible overestimate of surface area in 3D for a convex shape when voxelized. The voxel mesh overestimates surface area by 50%, and the overestimate is reduced to around 16% using the structured tetrahedral smoothing method proposed in this paper. Previous work [29] considered a circle in 2D. For the 2D circle (analogous to an infinite cylinder) the surface area overestimate dropped from 20% to 5% using the smoothing methodology. It is expected that other objects would demonstrate surface area overestimates between the cylinder and sphere. This is observed when investigating surface areas for the four tumors in Figure 11(c) and Figure 11(d). Each of the tumors’ surface-area ratios show a steady drop from about 47% to about 17%, which lies between the overestimations for the cylinder and sphere.

The effect of an overestimation of surface area for heat-transfer simulation is shown with the example of a sphere. Figure 12 shows the radial temperature plot for the sphere. The voxel mesh, having the highest overestimation of surface area, results in the most heat loss from the sphere and the lowest surface temperature. The tetrahedral smoothed domain reduces this error by around 50%. The temperature error calculated from Equation (8) is also shown in Figure 12 . For the voxel sphere, the surface area overestimated by about 50%, and the temperature error obtained is 0.55 °C. This surface error is reduced to around 18%, and the temperature error is 0.25 °C. The heat transfer simulation for a 2D circle is shown in previous work [29]. Since the smoothing method doesn't affect a flat boundary or surface, no change would be observed in area on two ends of a finite cylinder. Using Equation (8), it is shown that the error in the temperature for a sphere is independent of its radial position and is a constant value. Similarly, it can be seen that the temperature error in a cylinder with adiabatic boundary condition at two ends will be independent of the radial position. A detailed simulation with different boundary conditions for a cylinder is beyond the scope of this paper.

The heat transfer simulation results for tumor 1 are shown in Figure 13. The figure compares the temperature map of a voxel unsmoothed and tetrahedral smoothed tumor at a cross-section taken at midpoint of the *z*-axial length. Referencing Figure 11, the volume of the voxel domain is more than the NURBS reference for the tumors. This is due to the resolution of the MRI scan. A finer resolution would result in better volume convergence of the voxel to the actual domain. As the voxel resolution increases, the amount of volume lost during the removal method decreases, and the volume difference is negligible.

At the voxel resolution used for the tumor simulations, there is a 4% reduction in the volume of the tumors after application of the proposed method. Since the voxel tumor volumes were already greater than the NURBS tumor volumes (used as reference), the reduction in volume results in a better accuracy of volume and surface area with respect to NURBS tumors. The voxelized tumors thus show a higher core temperature compared to a smoothed tumor due to the volume difference. This difference in maximum temperatures for all four tumors is within 0.01 °C for the simulations conducted. The thermal maps for the remaining three tumors studied are shown in Figure 14, Figure 15 and Figure 16 in supplementary data section. These three tumors show a similar temperature profile as that observed in Figure 13. As stated previously, when voxel dimensions are decreased further, this difference in volume becomes negligible and only changes in surface area are observed. Execution of the smoothing method and heat transfer simulation for the tumors was used to demonstrate applicability of the proposed methodology on a domain directly generated from MRI data. Thus, it can thus be applied to any animal or human using medical imaging data of the subject.

To maintain simplicity for this study, the perfusion rate of the blood flow was taken as zero and the assumption of no other heat source acting on the domain under study except metabolic heat was made. The study was performed for a system under steady state. Coupled with the assumed boundary conditions, these assumptions reduced the Pennes Bioheat Equation to a simple conduction heat equation with heat generation term as shown in Equation (2). If these assumptions are not made, Equation (1) could have been discretized using the forward timestep approach as shown in Equation (9)

\begin{align*}
&{\rho _T}{c_T}\frac{{\left({T_0^{j + 1} - T_0^j} \right)}}{{{{\Delta }}t}}\text{d}V = \mathop \sum \limits_{i = 1}^n {\left({UA} \right)_i}\left({T_i^j - T_0^j} \right)\\
&\quad + {\omega _b}{c_b}\text{d}V\left({T_b^j - T_0^j} \right) + \dot q\text{d}V \tag{9}
\end{align*}

The superscript *j* represents the time step and ${{\Delta }}t$ represents the time step difference. For steady state, if the perfusion term is non-zero, the above discretization method can still be applied to determine the temperature profile of the domain. However, the allowable volume error would need to be reassessed since the amount of blood perfused depends on the net volume of tissue in Pennes Bioheat Equation. Thus, an error in the volume of domain due to removal method discussed in this paper, will result in an error in the total blood perfused in the domain. This error in the net blood perfused compared to expected net blood perfusion will contribute to the error in temperature in addition to the error originating from surface area and volume differences. The discretization adopted in this paper allows the properties of domain to vary from one mesh element to another. Thus, the method could still be used if the surrounding healthy tissue assumed in this paper had a different thermal conductivity than that of the tumor.

As discussed in the thermophysiological model section of this paper, skin temperature and core temperature are inputs for the hypothalamus to provide response signals. Accuracy in these values is important when simulating the human thermoregulatory mechanism. Surface area error results in reduced accuracy of skin and core temperatures. Reduction of surface area error from 50% to 15% for sphere and 47% to 17% for tumors is significant but requires more work for higher accuracy. The surface area error is much smaller when modeling the domain with a polygon mesh or unstructured mesh, since these techniques, including methods such as lattice cleaving [35], can be used to represent the curve with higher accuracy. However, these techniques present other challenges in terms of modeling bioheat transfer, especially for the domain of the entire human body. Since the goal is to extend this work to such a domain, a direct technique to generate models from medical imaging data and to maintain approximate representation is important. In a simple case such as the sphere, the actual surface area is known and thus overestimation of area can be calculated. When the domain is generated from medical imaging data, actual surface area and volume are not known. The proposed methodology can be used to represent an individualized organ of any shape, or an entire human body, and perform simulations with better surface-area accuracy than can be achieved with voxels.

## VI. Conclusion

Human thermophysiological modeling still relies on stylized phantoms to model the human anatomy. Parallel to this field of research, computational human phantoms have been developed that are more advanced and detailed compared to stylized phantoms. Despite the availability of these phantoms, they are not being used in human thermophysiological research. This paper examines the challenges of stair-step effect, which is faced when using a voxel phantom to model a human body for thermoregulation.

The stair-step effect of the voxel domain is addressed by introducing a methodology to generate a structured mesh from medical imaging data in a manner similar to lattice cleaving. Application of this methodology is demonstrated by MRI scans of four mice tumors and a generic sphere. The methodology proposed has been shown to reduce the overestimation error from 50% to 15% for a generic sphere and 47% to 17% for tumors generated from MRI scans.

The surface area overestimation results in error in temperature of the domain for heat transfer simulation. This error is reduced using the tetrahedral smoothing method discussed in this paper. This methodology, while not perfect, provides a direct approach to convert medical imaging data to a tetrahedral structured mesh. Further study to better understand how different cuts in a voxel can be performed while maintaining the structured mesh can help to reduce this error further. For example, in this paper, all the cuts in the voxel were made passing through the centroid of the voxel. Thus, all the tetrahedrons generated were of same size. If the cuts are made offset to the centroid, tetrahedrons of different sizes and angles can be generated to smoothen the surface more.

The technique is independent of domain material and can be extended with ease to non-homogeneous structures. Similar to the tumors, the entire human body can be generated from MRI or computed tomography scans and smoothed. This allows the simulation domain to be individualized. Generating the entire human body from medical imaging data and applying heat transfer simulation to it with control mechanisms for thermoregulation would result in a more complete simulation for human thermophysiological modeling.

Future work consists of extending the simulation to the entire body and adding the blood flow network. A heat transfer solver coupled with the blood flow network will provide a human thermal comfort model. Additionally, in this study, the voxels were revoxelized to isotropic voxels. A study to understand the variation of volume and surface area when the voxel dimensions are anisotropic (i.e., different in two or three dimensions) is being conducted and will be presented in the future work.

## Supplementary Materials


