# Supplementary material for: A Structured Cleaving Mesh for Bioheat Transfer Application
Source: IEEE Open J Eng Med Biol. 2020 May 14;1:174–86. doi: 10.1109/OJEMB.2020.2994557 (PMC8974664; doi:10.1109/OJEMB.2020.2994557)
Supplement: Supplementary file 1 [file supp1-2994557.pdf]

Supplementary Materials

A Structured Cleaving Mesh for Bioheat Transfer Application

Rohan Amare*, *Member, IEEE*, Amir A. Bahadori, *Member, IEEE* and Steven Eckels

The supplementary material presented here consists of cross sections and thermal maps for tumors shown in Figure 10.

Figure 14.a, Figure 15.a and Figure 16.a show the cross sections of tumor 2, 3 and 4 which are shown as NURBS objects in Figure 10.b, Figure 10.c and Figure 10.d respectively. The tetrahedral smoothed tumors are shown in Figure 10.f, Figure 10.g and Figure 10.h respectively. The thermal maps at these cross sections are shown for voxel unsmoothed tumors in Figure 14.b, Figure 15.b and Figure 16.b and tetrahedral smoothed tumors in Figure 14.c, Figure 15.c and Figure 16.c respectively.

The temperature contours for all tumors show that the voxel unsmoothed tumor has a higher core temperature than the tetrahedral smoothed tumors. This is due to the volume difference between the voxel unsmoothed and tetrahedral smoothed tumor. Figure 11 shows the volume and surface area convergence as a function of size of voxels used. A voxel size of 0.12 mm x 0.12 mm x 0.12 mm was used to simulate the temperature distributions shown.

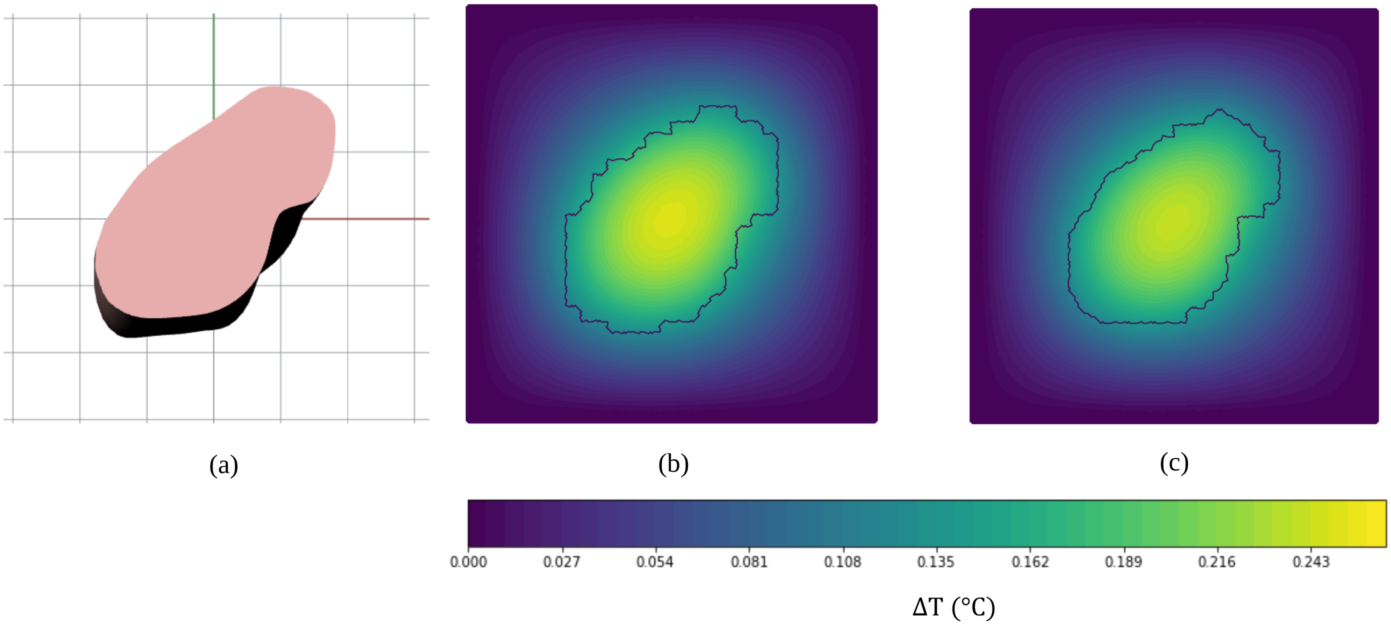

Figure 14: Cross-section of tumor 2 at x-y plane at midpoint of z-axial length
(a) NURBS Rhinoceros model (b) voxel unsmoothed (c) tetrahedral smoothed.

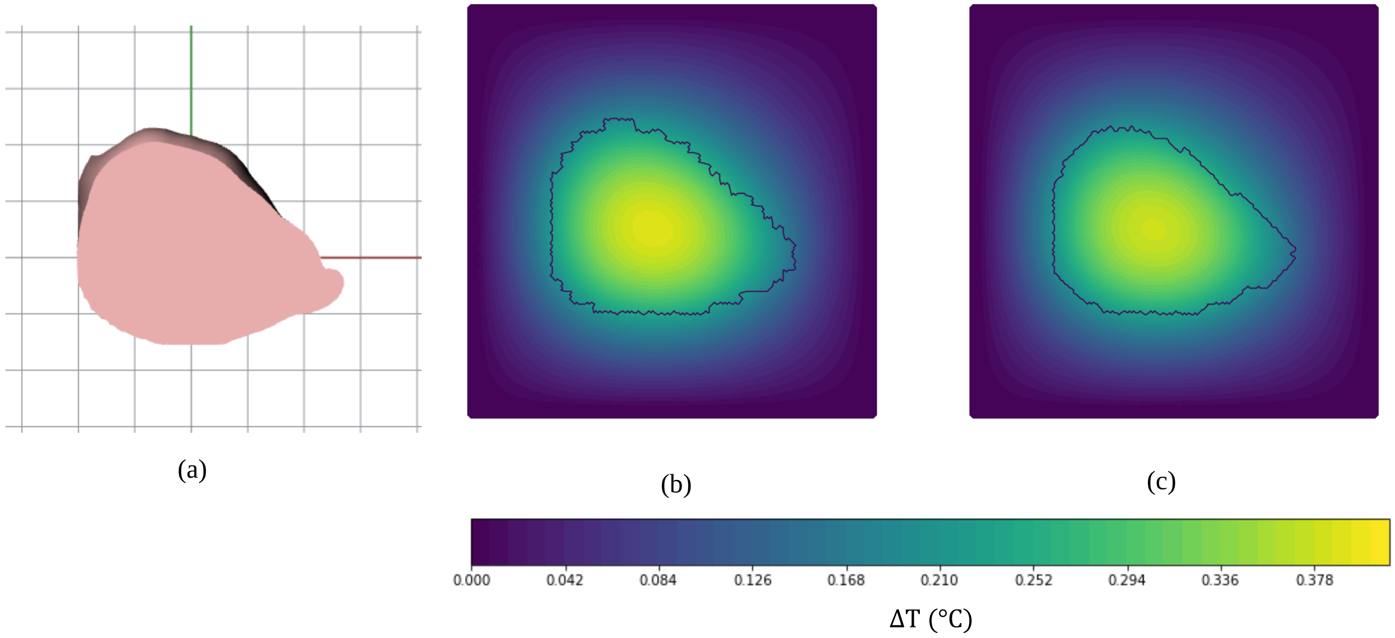

Figure 15: Cross-section of tumor 3 at x-y plane at midpoint of z-axial length
(a) NURBS Rhinoceros model (b) voxel unsmoothed (c) tetrahedral smoothed.

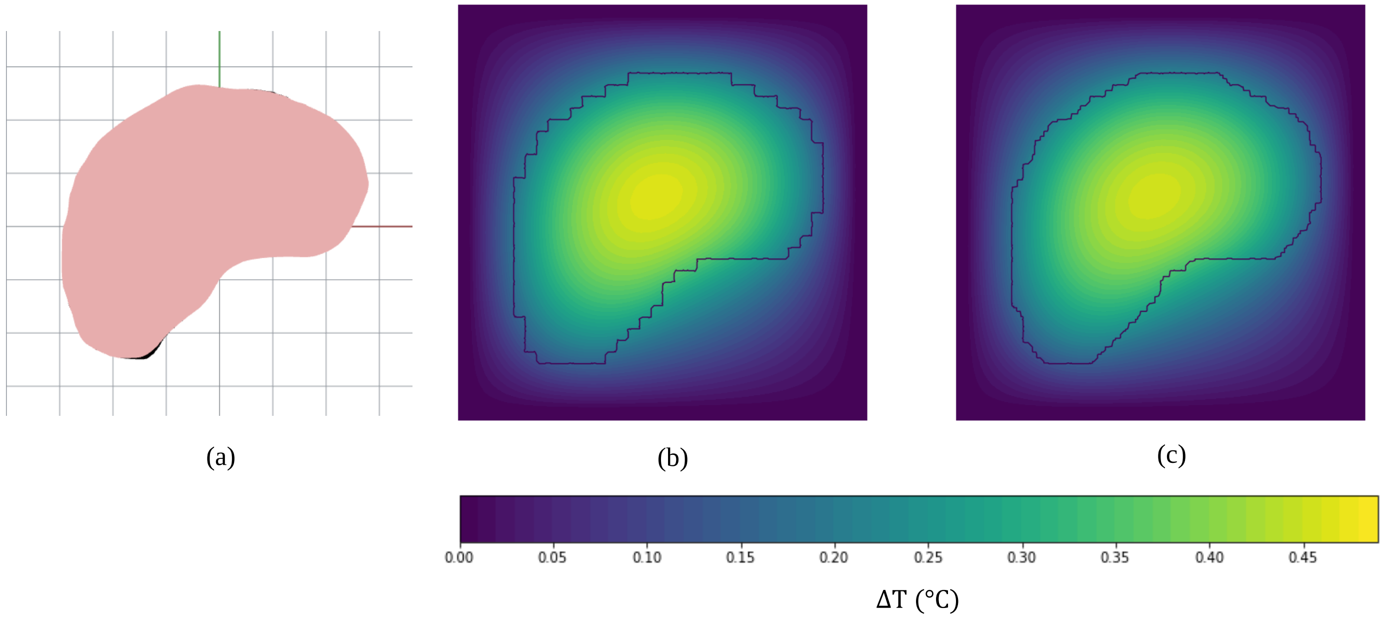

Figure 16: Cross-section of tumor 4 at x-y plane at midpoint of z-axial length
(a) NURBS Rhinoceros model (b) voxel unsmoothed (c) tetrahedral smoothed.
